# Sclerosing Angiomatoid Nodular Transformation of the Spleen: A Report of Rare Case and Literature Review

**DOI:** 10.7759/cureus.45422

**Published:** 2023-09-17

**Authors:** FNU Raja, Vinesh Kumar, Eric Moll, Azzam Hammad, Salman Ayub

**Affiliations:** 1 Pathology, MetroHealth Medical Center, Cleveland, USA; 2 Pathology, Metrohealth Medical Center, Cleveland, USA

**Keywords:** fine needle aspiration (fna), treatment therapy of sant, immunohistochemistry, splenectomy, sclerosing angiomatoid nodular transformation

## Abstract

Sclerosing angiomatoid nodular transformation (SANT) is a benign vascular lesion of the spleen with uncertain etiology. It predominantly affects women between the ages of 30 and 60 years. Clinically, it is asymptomatic or can cause abdominal pain, but usually discovered incidentally on imaging, which can identify a mass but may not provide a definitive diagnosis. In uncertain vascular lesions, there is always a risk of spontaneous rupture of large vessels and the potential for spreading malignancy. Hence, the final diagnosis is rendered on microscopy after splenectomy. A middle-aged female came to the clinic complaining of abdominal pain. Radiology showed a solid splenic mass and the patient underwent splenectomy. Gross examination showed a 3 cm white firm mass with focal hemorrhage. Microscopy revealed multiple nodules of variable sizes surrounded by fibrosclerotic stroma. The nodules showed round to slit-like vascular spaces with numerous red blood cells. The internodular stroma consisted of dense fibrous tissue with scattered plump myofibroblasts and lymphoplasmacytic inflammatory cells. These distinctive features lead to the diagnosis of SANT. SANT possesses characteristic histologic features with distinctive immunohistochemistry (IHC). IHC reveals three different types of vessels within the nodules as follows: (1) small veins (CD34^-^, CD31^+^, CD8^-^), (2) sinusoids (CD34^-^, CD31^+^, CD8^+^), and (3) capillaries (CD34^+^, CD31^+^, CD8^-^). All three types of vessels are negative for CD21/CD35 and CD68. Hemangioma and littoral cell angioma are two frequent vascular tumors in the spleen that should be considered differential diagnoses. Both lesions lack the microscopic features of SANT and have only a single type of vessel. The vessels in hemangioma are (CD31^+^, CD34^+^, CD8^-^), while in littoral cell angioma they are (CD31^+^, CD34^-^, CD8^-^, CD21^+^, CD68^+^). There are no specific clinical or radiologic findings for SANT. It is important to recognize these characteristic features and to differentiate them from other benign and malignant lesions, such as angiosarcoma. A thorough histopathologic examination and IHC are helpful in making the correct diagnosis.

## Introduction

Sclerosing angiomatoid nodular transformation (SANT) is a rare benign vascular lesion of the spleen. Its exact pathogenesis is still uncertain [1]. Commonly, it occurs during middle age, between the ages of 30 and 60, with a higher prevalence among females [2]. Clinically, it is asymptomatic and usually discovered incidentally on imaging, but can cause non-specific abdominal discomfort or pain. It is a proliferative lesion that can grow up to 17.0 cm in the greatest dimension [3].

A radiology workup can identify a mass lesion but cannot provide a definitive diagnosis. Ultrasonography reveals a hypoechoic mass while a CT scan reveals a heterogeneous, low-attenuation lesion in the spleen [4]. Occasionally, due to their large size, these lesions can simulate a primary malignant or metastatic lesion. It is quite challenging to diagnose SANT before surgery as there is a lack of sensitivity and specificity in radiology, and there are no specific serologic markers available. In uncertain vascular lesions, there is always a high risk of spontaneous rupture of large vessels and the potential for spreading of malignancy on performing fine needle aspiration (FNA).

Consequently, the current standardized approach for diagnosing this vascular condition relies on histopathologic examination after surgical removal. Open or laparoscopic splenectomy is currently the preferred treatment for SANT to avoid the risk of spontaneous vessel rupture and to rule out the suspicion of malignancy [5]. Although SANT is typically confined to the spleen, there has been a reported case involving the adrenal gland [6].

In this report, we present a rare case of asymptomatic SANT in the spleen of a middle-aged woman. The incidental splenic mass was successfully treated with laparoscopic splenectomy and confirmed through postoperative diagnosis. Additionally, we provide a literature review on SANT to enhance understanding of this condition when encountering a splenic tumor.

## Case presentation

**Figure 1:**
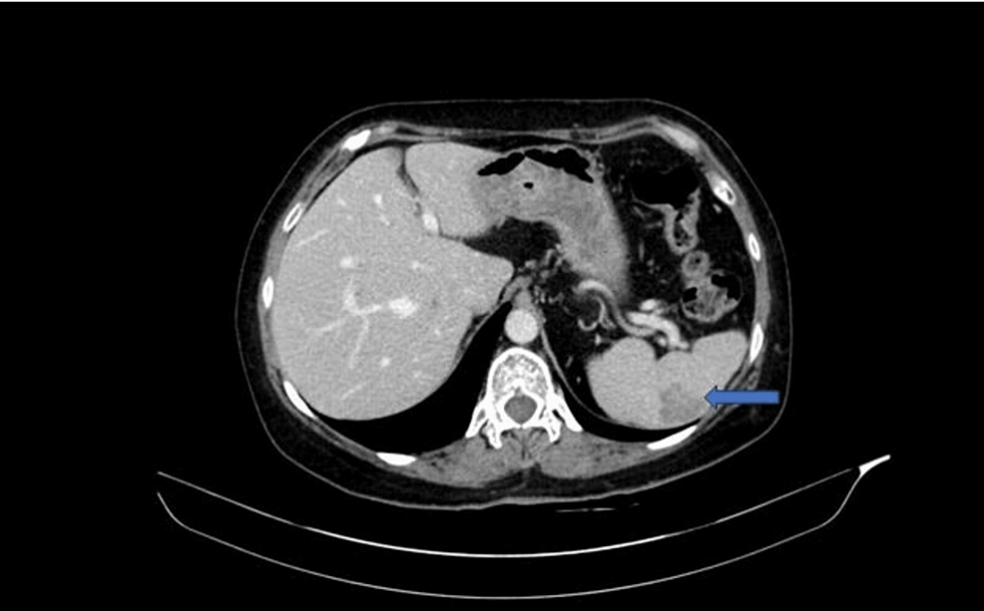
CT abdomen/pelvis with contrast indicates a hypodense mass lesion in the spleen (arrow).

A middle-aged woman with a medical history of diabetes mellitus underwent an abdominopelvic CT scan for an unrelated condition. The scan incidentally revealed a hypodense mass lesion in her spleen, measuring 1.7 cm in its largest dimension. Over a span of four years, the mass grew from 1.7 cm to 3.4 cm (Figure 1). Her blood work showed no significant abnormalities, except for mild normocytic anemia, with a hemoglobin level of 11.4 g/dL. The patient underwent a robotic-assisted partial splenectomy to remove the mass.

**Figure 2:**
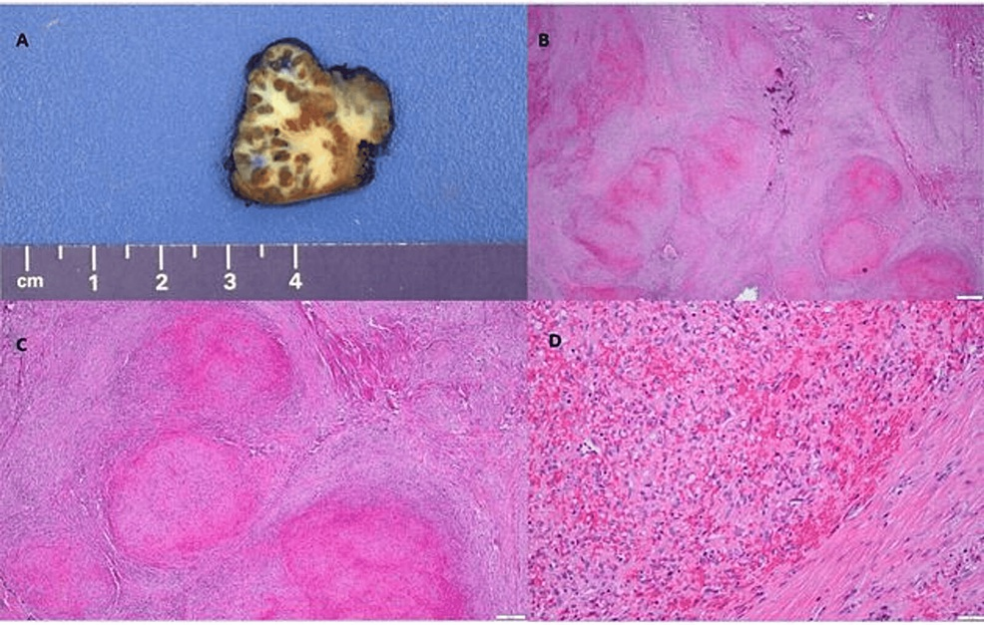
The examination of the specimen indicating the presence of a tumor of the spleen. Gross image of the lesion (A); hematoxylin-eosin stain, objectives 2× (B), 4× (C), and 20× (D).

The gross examination of the specimen indicated the presence of a tan-brown, rubbery portion of the spleen, weighing 8.5 g, and measuring 3.5 cm. The outer surface appeared smooth and shiny, with no identifiable nodules. Upon making cut sections, a white-tan sclerotic mass with focal hemorrhage was observed, measuring 3.4 cm (Figure 2A). The microscopic examination at low power revealed the presence of multiple nodules of different sizes, encompassed by fibrosclerotic stroma (Figure 2B). On closer examination at higher magnification, the nodules displayed round to slit-like vascular spaces containing numerous red blood cells. The internodular stroma consisted of dense fibrous tissue with scattered plump myofibroblasts and lymphoplasmacytic inflammatory cells (Figures 2C, 2D). These unique and specific morphological characteristics led to the diagnosis of sclerosing angiomatoid nodular transformation (SANT) of the spleen. At five months follow-up, there have been no recurrences of the symptoms.

## Discussion

SANT is characterized as a vascular proliferation occurring in the red pulp of the spleen, forming multiple sclerotic nodules. The precise pathogenesis of this condition remains not entirely understood. Some authors propose that SANT might represent the end-stage of benign splenic conditions, such as capillary hemangioma or hamartoma [2].

On the other hand, other studies have suggested a potential association with Epstein-Barr virus (EBV) infection, leading to some calling it an inflammatory pseudotumor. Additionally, there have been links made between SANT and immunoglobulin G4-related autoimmune diseases [7,8]. However, in 2004, Martel et al. conducted a study involving 25 cases of this lesion and introduced the term SANT to describe this specific type of condition [1].

Initially, it was suggested that SANT commonly occurs during middle age, with a predilection for females. However, as more cases have been reported and studied, this female preponderance may have decreased [9].

Diagnosing SANT preoperatively remains a challenging task. Radiology workup can offer valuable clues that help narrow down potential differentials. CT scans often depict the lesion as a hypodense mass with a well-demarcated border and peripheral nodular enhancement, which suggests the presence of a vascular rim. MRI typically shows centripetal and progressive enhancement of the lesion.

These radiological findings may provide indications that the lesion represents a non-malignant vascular type, such as hemangiomas. However, it is important to note that similar imaging characteristics can also be observed in malignant lesions, such as angiosarcoma. Therefore, despite the valuable insights from radiology, histologic examination remains the gold standard method for diagnosing such rare lesions [10].

During the initial histopathologic examination conducted by Martel et al. and several other researchers, they identified three characteristic and distinct types of blood vessels within the lesion [1]. The first type comprises well-formed cord capillaries arranged in a lobular pattern. Immunohistochemistry (IHC) testing revealed positivity for CD34 and CD31, while CD8 showed a negative result. The second type consisted of splenic sinusoids, and IHC staining demonstrated positivity for CD68 and CD31, while CD34 was negative. The third type involved small veins exhibiting a mesh-like pattern. On IHC, these veins showed positivity only for CD31, while CD34 and CD8 were negative.

These three distinct types of small blood vessels closely resembled the normal composition of the red pulp in the spleen. Importantly, D2-40 staining was negative in the endothelial cell linings, providing further confirmation of the lesion's vascular origin instead of being of lymphatic origin.

Identifying vascular lesions of the spleen, including SANT, can be challenging due to several other lesions with similar morphological and immunohistochemical characteristics. These lesions span a spectrum from benign to malignant. Among the benign lesions are littoral cell angioma, hemangioma, lymphangiomas, and hamartoma. On the other hand, the malignant category includes angiosarcoma.

Littoral cell angioma is a benign vascular type of lesion of the spleen that arises from the littoral cells. These cells line the splenic sinuses and perform a role in hemophagocytosis [11]. Microscopic features demonstrate an anastomotic vascular channel lined by tall columnar cells either in a flat or papillary configuration. The cells show positive reactivity for endothelial and histiocytic markers [11]. On IHC, these cells are positive for CD68 and CD21, while negative for CD34 and CD8. Morphology and IHC help to differentiate them from SANT.

Splenic hemangioma, arising from sinusoidal endothelial cells is the most prevalent benign lesion found in the spleen [12]. It can manifest as either localized or diffuse and may exhibit either a cavernous or capillary configuration. Histologically, the lesion is characterized by proliferating vascular channels separated by thin, fibrous septa and lined with flat endothelial cells. In some larger lesions, thrombosis, infarction, and pseudocystic degeneration can be present [13]. The vascular endothelial cells in splenic hemangiomas exhibit positive immunoreactivity for vascular endothelial markers CD31 and CD34. Conversely, they do not show immunoreactivity for CD8, CD21, and CD68. It is worth noting that diffuse splenic hemangiomas may display CD68 immunoreactivity in some cases; however, these lesions do not exhibit the characteristic trivascular pattern of proliferation observed in SANT.

Lymphangioma is a benign tumor affecting the lymphatic endothelium in the spleen. It can manifest as either a single nodule or as a diffuse process. Most of the cases present cyst formation, but some cases show capillary and cavernous patterns. Histologic examination reveals cystic spaces lined by a single layer or multiple layers of endothelial cells. The cystic spaces are filled with pinkish proteinaceous material. Notably, apart from the conventional endothelial markers, the lymphatic endothelium exhibits strong immunoreactivity for D2-40 while being negative for CD21 and CD8 [12].

Splenic hamartoma (SH), also known as splenoma, is a rare vasoformative lesion of the spleen arising from the cells lining the splenic sinuses. Under microscopic examination, it is characterized by predominantly disorganized red pulp elements with limited fibrous trabeculae. There may be varying amounts of extramedullary hematopoiesis as well. Unlike in some other lesions, splenic hamartoma typically lacks lymphoid follicles and dendritic cells. IHC reveals positive expression for factor VIII, CD31, CD8, and type IV collagen, while CD21 and CD68 show negative results. Distinguishing features between SH and SANT include the absence of a nodular growth pattern in SH and the presence of the classical immunophenotype in SANT [14].

Angiosarcoma stands as the most prevalent non-lymphoid malignant primary tumor affecting the spleen [13]. Histologically, it exhibits irregular and anastomosing vascular channels, characterized by significant cytologic atypia, brisk and high mitotic count, and invasion. The atypical cells show a vascular endothelial phenotype, with some positivity for CD68. The key distinguishing factors between angiosarcoma and splenic angiomatosis with SANT include the absence of a nodular growth pattern and the presence of invasion, cytologic atypia, and mitosis in angiosarcoma [15].

In terms of management and prognosis, SANT behaves in a clinically benign manner and has a favorable prognosis. To date, no recurrences have been reported. The gold standard treatment is splenectomy. However, during the surgical procedure, caution is exercised to avoid morcellation of the spleen. This approach is taken due to the uncertain nature of the lesion's diagnosis and to prevent the seeding of potential malignancy. Lastly, it is significant to provide proper vaccination after splenectomy to prevent infection, especially against encapsulated bacteria.

## Conclusions

To the best of our knowledge, there are approximately 170 cases described in the literature. As of now, there are no specified clinical-radiologic findings. It is far more significant for the pathologist to recognize this rare lesion. The distinct morphologic features, along with IHC differentiate it from other non-malignant or malignant lesions.
